# Protozoan Cysts in Faecal Pellets of German Cockroaches (*Blattella germanica*), with Particular Emphasis on *Lophomonas blattarum*

**DOI:** 10.2478/s11686-020-00213-2

**Published:** 2020-05-29

**Authors:** Hugo Cornelis van Woerden, Rafael Martínez-Girón, Cristina Martínez-Torre

**Affiliations:** 1 Centre for Health Science, University of the Highlands and Islands, Old Perth Road, Inverness, IV2 3JH UK; 2 Protozoal Respiratory Pathology Research Unit. INCLÍNICA Foundation, Calvo Sotelo 16., 33007 Oviedo, Spain; 3 Faculty of Pharmacy, University of Salamanca, Campus Miguel de Unamuno. Lic. Méndez Nieto, s/n, 37007 Salamanca, Spain

## Abstract

**Purpose:**

The aim of this study was to investigate protozoan cysts and oocyts in the faecal pellets of the German cockroach (*Blattella germanica*), with emphasis on the prevalence of *Lophomonas blattarum*.

**Methods:**

Thirty adult *Blattella germanica* (12 males; 18 females) were trapped near Madrid, Spain. Expelled faecal pellets were collected in the laboratory over 5 days in two plastic containers. Protozoan cysts from one container were weighted and those in the other used for morphologically identification.

**Results:**

Protozoan cysts/oocysts per gram of faecal pellet were: *Nyctotherus* sp. (0.0019/g), *Entamoeba* (0.0007/g), *Balantidium coli* (0.0001/g), *Lophomonas blattarum* (0.00038/g). Observation of 189 protozoan cysts/oocysts indicated the following prevalence: *Nyctotherus* sp. 65 (34.4%); *Gregarina* spp. 34 (18%); *Entamoeba* sp. 24 (12.7%); *Cryptosporidium* sp. 17 (9%); Coccidia 16 (8.4%); *Lophomonas blattarum* 13 (6.8%); *Balantidium coli* 4 (2.1%); and unclassified 16 (8.4%).

**Conclusion:**

*Lophomonas blattarum* has previously been demonstrated in the gut of cockroaches, but this is the first assessment of the prevalence in *Blattella germanica* in faecal pellets. The presence of protozoa in faecal pellets provides some evidence for one step in a hypothesised route of respiratory infection whereby protozoa from household insects enter the respiratory tract.

## Introduction

This paper examines one step on a hypothesised pathway, whereby protozoa from cockroach intestine are excreted in encysted form in faecal pellets, consequently present in household dust, and then inhaled into the human respiratory tract. Inhaled protozoa might subsequently be expected to excyst and cause commensal or pathogenic infection of the respiratory system.

Cockroaches are a group of arthropods that are closely associated with an anthropogenic environment, which can adversely impact on human health. The adverse effect that has been best evidenced is allergy to cockroach proteins (mainly proteases) present in its body, in saliva, intestinal contents, or in its faecal pellets, which are associated with asthma in some patients [1]. There is also evidence for the role of cockroaches as a reservoir for pathogenic microorganisms [2]. The German cockroach (*Blattella germanica*), can carry medically important parasites on its surface and inside its digestive tract, including protozoa such as *Ballantidium coli*, *Blastocystis hominis*, *Entamoeba histolytica*, *Giardia duodenalis*, and *Toxoplasma gondii* [3, 4]. We have similarly described the presence of a multi-flagellated protozoon, *L. blattarum,* in the gut of *B. germanica* [5].

Protozoa have a number of mechanisms for ensuring survival in adverse environment conditions. Relevant adverse environmental conditions include: lack of nutrients, increased osmotic pressure, temperature changes, low pH, and internal accumulation of waste products. In such circumstances, many protozoa form resistant cysts [6]. During this process (encystation), the cytoplasm is surrounded by a rigid or semi-rigid wall. The cyst wall is secreted by the organism. Thus, by means of a cystic form, protozoa can survive in the environment and transmit from one host to another. When a mature cyst enters a suitable host, excystation occurs and the excysted trophozoite of the relevant protozoal species can begin a new period of growth and reproduction.

Relatively few studies have assessed the presence of protozoa in the faecal pellets of cockroaches, but a number of studies of protozoa in cockroaches have examined the external body surface and/or the intestinal contents [7–10]. A separate line of research has identified allergenic components in faecal pellets [11–13], but the presence of microorganisms, including protists, has not been extensively researched in cockroach faecal pellets [14].

Our interest in *L. Blattarum* is based on two factors: first, the potential relationship between this protozoan and broncho-pulmonary disease [15], and second, our observation of both the excystation of this protozoon and the presence of immature trophozoites in human respiratory secretions [16]. *L. Blattarum* is a multi-flagellated protozoon, which is round to ovoid in shape (20–60 µm in diameter), with a double tuft of flagella inserted at the anterior end, and containing coarse granules and some phagocytic vacuoles in the cytoplasm.

The aim of this study was, therefore, to analyse faecal pellets from the German cockroach (*B. germanica*) to observe and confirm the presence of protozoan cysts, quantify the count per faecal mass, and use morphological features to catalogue observed protozoa, with specific emphasis on *L. blattarum* cysts.

## Materials and Methods

**Fig. 1:**
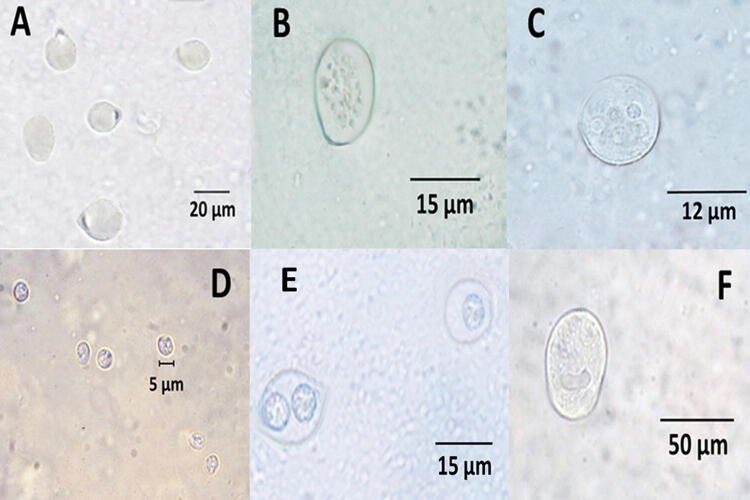
Different types of protozoal cysts and oocysts observed in fresh samples from faecal pellets. **a** *Nyctotherus* sp. (× 1000). **b** *Gregarina* spp. (× 1000). **c** *Entamoeba* sp. (× 100). **d** *Cryptosporidium* sp. (× 1000). **e** Coccidia (× 1000) **f** *Balantidium coli* (× 1000)

In the summer of 2018, cockroach trapping was undertaken in a domestic environment near Madrid, Spain. To achieve this, a modified plastic bottle was used, containing an attractant, which was composed of damp bread, sugar and potato. Once captured and transported to the laboratory, the cockroaches were transferred to two clean plastic tapered boxes, with holes punched in the plastic to allow for air flow, and with filter paper as a base. A total of 30 adults (12 males and 18 females) belonging to the species *B. germanica* were caught. The boxes containing the cockroaches were placed in a dark cabinet in the laboratory and stored at room temperature (21 °C) and 77% humidity. The food provided was pieces of biscuit, banana and moist bread. After 5 days in captivity, expelled faecal pellets (which were dark, cylindrical in shape and ranging between 0.5 and 1.5 mm in size) were carefully collected with the help of a magnifying glass, thus avoiding any contamination with the remains of cockroach body parts (Fig. 1). Faecal pellets obtained from one of the plastic boxes were used to provide a cyst count, whilst those obtained from the other box were used for morphological characterization of the cysts.

At the end of five consecutive days in captivity, the weight of faecal pellets collected was 0.3402 g. For the first analysis, the cockroach faecal pellets from one box were gently crushed and mixed with a saturated salt solution (NaCl 400 g and distilled water 1000 ml) in preparation for cyst counting. The resulting mixture was filtered and placed in a McMaster egg slide counting chamber with two grids and assessed under a light microscope (40 × and oil immersion objective).

For the second analysis, to allow morphological identification under light microscopy, the faecal pellets from the other box were gently crushed with the tip of sterile tweezers and the resulting material was mixed and diluted with a saline formaldehyde solution (SFS) composed of 950 ml of distilled water, 5 g of sodium chloride and 50 ml of formaldehyde (37% solution) by the sedimentation method. The proportion of crushed faecal pellet to SFS was 1:9. The resultant mixture was filtered and placed in several centrifuge tubes, 2 ml of sulphuric ether (diethyl ether) was added, and the tubes were waved to allow mixing. The tubes were then centrifuged at 1000 rpm for 5 min. The supernatant was decanted and the sediment was picked up with a Pasteur pipette. The sediment was partitioned, placed onto glass slides, and examined under a light microscope using a 40 × oil immersion lens. Samples that were positive for cysts, mainly those that were classed as potentially *L. blattarum*, were stored in a plastic microscope slide box and subsequently stained with 1% Lugol´s Iodine and Wheatley´s trichrome (see method in supplementary file) to assist in confirmation of classification, particularly with reference to identification of *L. blattarum*. All cysts and oocysts were identified based on their morphology by comparison against standard taxonomic keys [17]. In addition, drawings made by Kudo of *L. blattarum* cysts [18], were particularly useful in cataloguing cysts of this multi-flagellated protozoon. Cryptosporidium oocysts were stained and identified using the trichrome stain. Cryptosporidium oocysts were rounded, measured 4–5.4 µm in diameter and displayed an internal granular-looking material with a mauve hue. They also appeared as unstained structures under oil immersion.

After the examination of the faecal pellets, the captured cockroaches were dissected to allow examination of their digestive tract to determine the proportion with *L. blattarum.* Each specimen was first anesthetized in a laboratory glass jar using cotton impregnated with ether. Cockroaches were exposed to the anaesthetic agent for 15–30 min. Full anaesthesia was checked by ensuring that their bodies did not respond to tactile stimuli. The insects were subsequently killed by decapitation. After this, dissection was carried out under a binocular lens. The midgut and hindgut were removed and sectioned, and the intestinal extracts were placed onto glass slides for microscopic observation of the fresh intestinal contents. In total, the cockroaches were kept for 10 days in captivity (six insects were sacrificed each day).

## Ethical Statement

This research was conducted humanely, in line with the “Guidelines for Ethical Treatment of Animals in Applied Animal Behaviour and Welfare Research” that have been developed by the International Society for Applied Ethology.

## Results

In a first analysis, faecal pellets were examined using a McMaster chambers under the light microscope, a total of 237 counting elements were identified. The number of counting elements and protozoan cysts/oocysts in faecal pellets was 0.00069 elements per gram and 0.00055 cysts per gram respectively. *Nyctotherus* sp. (0.00019/g), were the most frequently observed cysts and five types which belong to pathogenic microorganisms were found (*Entamoeba*, *Cryptosporidium*, Coccidia, *L. blattarum* and *Balantidium coli*), representing 0.00021 cysts per gram of faecal pellets. *Entamoeba* were the most frequent (0.00007/g) and *Balantidium coli* the least common (0.00001/g). *L. blattarum* cysts had a prevalence of 0.000038 cysts per gram of faecal pellets.

In a second analysis of the faecal pellet sediments, 189 protozoan cysts/oocysts belonging to the following types were identified (Fig. 1): *Nyctotherus* sp. 65 (34.4%); *Gregarina* spp. 34 (18%); *Entamoeba* sp. 24 (12.7%); *Cryptosporidium* sp. 17 (9%); Coccidia 16 (8.4%); *L. blattarum*-like 13 (6.8%); *Balantidium coli* 4 (2.1%); and unclassified 16 (8.4%). There were some gender differences, with 43 *Nyctotherus* sp. cysts observed in the 12 male cockroaches and 22 in the 18 females; similarly 9 *Gregarina* spp. cysts were observed in the 12 male cockroaches and 25 in the 18 females.

**Fig. 2:**
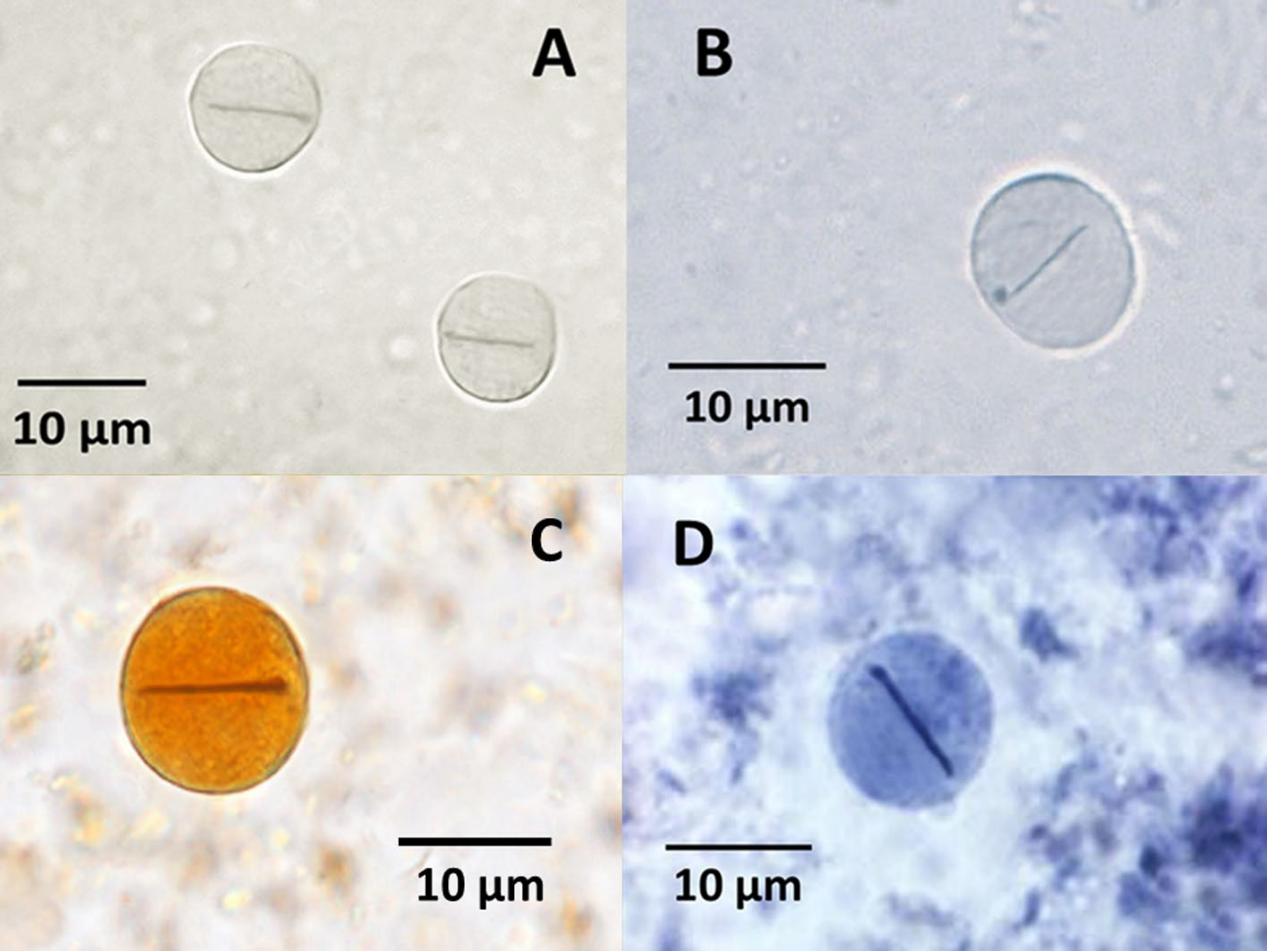
Lophomonas blattarum cyst from faecal pellets. **a**, **b** fresh samples (× 1000). **c** Lugol´s iodine stain (× 1000). **d** Weathley´s trichrome stain (× 1000)

Sixteen *L. blattarum* cysts were identified, based on a typical round-oval shape, 10–12 µm in size, with a distinctive dark line (perhaps a chromatoidal bar), marking their diameters (Fig. 2). Unlike other protozoa cysts such as *Giardia* and *Entamoeba*, the presence of nuclei was not observed.

**Fig. 3:**
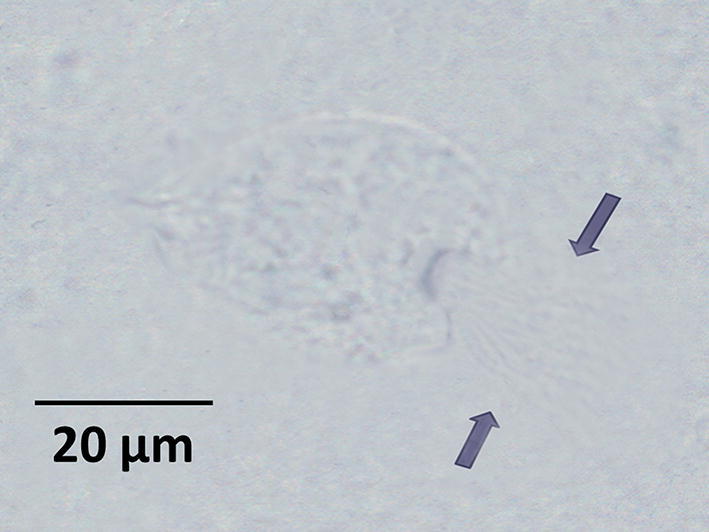
Trophozoite of *Lophomonas blattarum* in the intestinal extract from the gut of *Blattella germanica* in a fresh sample (× 1200)

Of the 30 captive cockroaches, 23 were alive after 1 week. The proportion of cockroaches containing free trophozoites of *L. blattarum* in fresh samples of intestinal extracts on dissection (Fig. 3) was 5/23 (16.6%).

## Discussion

The medical importance of cockroaches lies primarily within two domains: an allergic/inflammatory response to cockroach allergens [19], and its role as a reservoir and source of pathogenic microbes in, or close to, human dwellings [20].

Some recent data suggest that infestation of homes with cockroaches is increasing and the topic is, therefore, one of potential clinical importance [21]. As early as the second decade of the last century, Robert Hegner [22] recognised the potential relationship between cockroaches and protozoa. He recognised that cockroaches provide an entero-parasitic habitat and that their close proximity in human dwellings raised the possibility of the transmission of protozoa to human subjects. His paper refers to the presence of *L. blattarum,* among other flagellates, in cockroaches.

Cockroaches were also shown to play a role in the dissemination of the cysts of certain infectious protozoa in a study in Nigeria [23]. Their wider role constitutes the principal basis of the hypothesis underpinning piece of this work, specifically, that protozoa in cockroach faecal pellets can be inhaled into the human respiratory tract and cause commensal or pathogenic infection.

The difference observed between the two analyses, 0.00069 elements per gram and 0.00055 cysts per gram, may be attributed to the different solutions used for processing the faecal pellets, the presence of artefacts/contaminants, and the limitations of observation under light microscopy. A potential weakness of our study is that we did not have access to molecular methods for the characterising the different protozoa. Future studies would benefit from utilising such an approach. It should also be noted that an alternative approach to this project would have been to assess the number of organisms in each individual cockroach. It should also be noted that cockroaches were not kept separate between capture and microscopic examination and there is a small risk that transmission of protozoa could have occurred between different cockroaches. The study could also have been strengthened by repeated re-sampling of the cockroach population, for example, three separate repetitions of the study described in this paper would have allowed the calculation of more accurate mean and standard deviation values for protozoa and cyst prevalence.

In line with other studies [24, 25], *Nyctotherus* sp. were the most frequent protozoon found. This ciliated protozoon (which is non-pathogenic for humans) harbours methanogenic bacteria as endosymbionts, playing a major role in the hindgut metabolic activities of the cockroaches [26].

*L. blattarum* was found in the intestinal extracts of 16.6% of the cockroaches. The prevalence of *L. blattarum* in our previous study of 119 cockroaches (also *B. germanica*) was similar, at 13.6% [4]. A study in another species of cockroach (*Periplaneta americana*), observed *L. blattarum* in 40% of the intestinal extracts of 110 cockroaches [27].

The cockroach and termite digestive tract harbours a wide variety of microorganisms, including protozoa, which collaborate in the digestion of ligno-cellulose compounds [28]. It is well recognised that coprophagia, the ingestion of faecal pellets, is a mechanism to transmit and populate the hindgut of early instars nymphs of cockroaches [29] and may also be a source of transmission of protozoal cysts between generations of cockroach.

The role of cockroaches as possible vectors of human pathogens has widely been commented on, but their role in the transmission of pathogens has seldom been conclusively established. A wide range of papers mention cockroaches as potential mechanical disseminators of enteric protozoan pathogens including: *Entamoeba histolytica*, *Cryptosporidium parvum*, *Giardia duodenalis, Balantidium coli, Blastocystis hominis, Cyclospora spp., Endolimax nana, and Isospora beli* [6, 8, 30–32].

We have postulated that cysts may be inhaled and cause respiratory disease in humans [33]. Protozoal cysts, such as those of *E. histolytica,* were identified on the body surface of 17% of 430 American cockroaches [34], and inhalation of dust containing cysts of *E. histolytica* has been proposed as a possible route of primary pulmonary amebiasis [35, 36], as well as for *L. blattarum* [16]. Experimental mechanical transmission of *T. gondii* oocysts, *S. muris* sporocysts, and *T. canis* eggs from faecal pellets of cockroaches (*B. germanica* and *P. americana*) to mice and rats (as paratenic hosts) has been demonstrated in the laboratory [37–39].

In normal domestic conditions, cockroach faecal pellets are susceptible to desiccation. It is, therefore, feasible that cockroach faecal pellets may be broken down to form small particles, which are inhaled [40]. In a study on the measurement and characterization of airborne cockroach allergens during normal domestic exposure in homes, it was concluded that “airborne particles containing cockroach allergen (Bla g1) were mainly associated with particles > 10 μm”, and hence small enough to be inhaled [41]. The cysts observed in faecal pellets from cockroaches in our study ranged from 12 to 20 µm in diameter, suggesting that their aerosolisation and inhalation is feasible.

By way of comparison with other research, *L. blattarum* cysts were identified in two studies in centrifuged sediments of macerate intestinal extracts from the cockroach *P. americana* [42, 43], but few studies have focused specifically on the presence of *L. blattarum* cysts in faecal pellets in *B. germanica*.

It may be challenging to prove the potential role of cockroaches in the transmission of protozoa cysts to humans through their faecal pellets, but the proposed causal pathway merits ongoing investigation.

## Appendix 1: Wheatley’s Trichrome Procedure

First glass bucket: introduce the smears in ethanol 70% for 5 min.

Second glass bucket: Trichromic staining with Para-Pak Trichrome-Wheatley solution for 10 min.

Third bucket: decolouration (acidic alcohol: 4.5 ml of glacial acetic acid + 995.5 ml of ethanol 90%. Introduce the smears during 2–3 s.

Fourth bucket: introduce the smears in ethanol 96% (quick lavage).

Fifth bucket: introduce the smears in ethanol 100% for one min.

Sixth bucket: introduce the smears in ethanol 100% for 3 min.

Seventh bucket: introduce the smears in ethanol 100% during 5 min.

Eighth bucket: introduce the smears in Xylene during 1 min.

## References

[1] Rabito FA, Carlson JC, He H, Werthmann D, Schal C (2017). A single intervention for cockroach control reduces cockroach exposure and asthma morbidity in children. J Allergy Clin Immunol.

[2] Adenusi AA, Akinyemi MI, Akinsanya D (2018). Domiciliary cockroaches as carriers of human intestinal parasites in lagos metropolis, southwest Nigeria: implications for public health. J Arthropod Borne Dis.

[3] Hamu H, Debalke S, Zemene E, Birlie B, Mekonnen Z, Yewhalaw D (2014). Isolation of Intestinal parasites of public health importance from cockroaches (*Blattella germanica*) in Jimma Town.

[4] Oguz B, Özdal N, Orunc Kilinc Ö, Deger MS (2017). First investigation on vectorial potential of Blattella germanica in Turkey. Ankara Üniv Vet Fak Derg.

[5] Martínez-Girón R, Martínez-Torre C, van Woerden HC (2017). The prevalence of protozoa in the gut of German cockroaches (*Blattella germanica*) with special reference to Lophomonas blattarum. Parasitol Res.

[6] Bogitsh BJ, Carter CE, Oeltmann TN, Bogitsh BJ, Carter CE, Oetelman TN (2013). General characteristics of the Euprotista (Protozoa). Human parasitology.

[7] Chamavit P, Sahaisook P, Niamnuy N (2011). The majority of cockroaches from the Samutprakarn province of Thailand are carriers of parasitic organisms. EXCLI J.

[8] Pai HH, Ko YC, Chen ER (2003). Cockroaches (Periplaneta americana and Blattella germanica) as potential mechanical disseminators of Entamoeba histolytica. Acta Trop.

[9] Nedelchev S, Pilarska D, Takov D, Golemansky V (2013). Protozoan and nematode parasites of the American cockroach *Periplaneta americana* (L.) from Bulgaria. Acta Zool Bulg.

[10] Kinfu A, Erko B (2008). Cockroaches as carriers of human intestinal parasites in two localities in Ethiopia. Trans R Soc Trop Med Hyg.

[11] Yun YY, Ko SH, Park JW, Lee IY, Ree HI, Hong CS (2001). Comparison of allergenic components between German cockroach whole body and fecal extracts. Ann Allergy Asthma Immunol.

[12] Wu HQ, Liu ZG, Gao B, Li M, Ran PX, Xing M (2007). Localization of Per a 3 allergen in the gut and faecal pellets of the American cockroach (*Periplaneta americana*). Int J Immunogenet.

[13] Jeong KY, Kim CR, Park J, Han IS, Park JW, Yong TS (2013). Identification of novel allergenic components from German cockroach fecal extract by a proteomic approach. Int Arch Allergy Immunol.

[14] Kakumanu ML, Maritz JM, Carlton JM, Schal C (2018). Overlapping community compositions of gut and fecal microbiomes in lab-reared and field-collected German cockroaches. Appl Environ Microbiol.

[15] Martinez-Girón R, van Woerden HC (2013). Lophomonas blattarum and bronchopulmonary disease. J Med Microbiol.

[16] Martínez-Girón R (2015). Parabasalids in respiratory secretions and lung diseases. Chest.

[17] Corliss JO (2001) Protozoan Cysts and Spores. In: Encyclopedia of Life Sciences, John Wiley & Sons Ltd, Chichester.

[18] Kudo RR, Kudo RR (1954). Reproduction. Protozoology.

[19] Pomés A, Mueller GA, Randall TA, Chapman MD, Arruda LK (2017). New insights into cockroach allergens. Curr Allergy Asthma Rep.

[20] Memona H, Manzoor F, Anjum AA (2017). Cockroaches (Blattodea: Blattidae): a reservoir of pathogenic microbes in human-dwelling localities in Lahore. J Med Entomol.

[21] Nasirian H (2017). Infestation of cockroaches (Insecta: Blattaria) in the human dwelling environments: a systematic review and meta-analysis. Acta Trop.

[22] Hegner R (1929). The viability of paramecia and euglenae in the digestive tract of cockroaches. J Parasitol.

[23] Tatfeng YM, Usuanlele MU, Orukpe A, Digban AK, Okodua M, Oviasogie F, Turay AA (2005). Mechanical transmission of pathogenic organisms: the role of cockroaches. J Vector Borne Dis.

[24] Gijzen HJ, van der Drift C, Barugahare M, Op den Camp HJ (1994). Effect of host diet and hindgut microbial composition on cellulolytic activity in the hindgut of the American cockroach, Periplaneta americana. Appl Environ Microbiol.

[25] Morenikeji OA, Adebiyi A, Oluwayiose A (2016). Parasites in cockroaches recovered from residential houses around dumpsite in Ido local government area of Oyo state, Nigeria. ARRB.

[26] Gijzen HJ, Barugahare M (1992). Contribution of anaerobic protozoa and methanogens to hindgut metabolic activities of the American cockroach, Periplaneta americana. Appl Environ Microbiol.

[27] Yang JX, Tang YY, Fang ZM, Tong ZZ, Li YL, Wang T (2014). Investigation on Lophomonas blattarum infection in *Periplaneta americana* in Wuhan City. Chin J Parasitol Dis.

[28] Ohkuma M, Noda S, Hongoh Y, Nalepa CA, Inoue T (2009). Inheritance and diversification of symbiotic trichonymphid flagellates from a common ancestor of termites and the cockroach Cryptocercus. Proc Biol Sci.

[29] Bell WJ, Roth LM, Nalepa CA, Bell WJ, Roth LM, Nalepa CA (2007). Microbes: The unseen influence. Cockroaches. Ecology, behaviour, and natural history.

[30] Haile T, Mariam AT, Kiros S, Teffera Z (2018). Cockroaches as carriers of human gastrointestinal parasites in Wolkite Town, southwestern Ethiopia. J Parasitol Vector Biol.

[31] Atiokeng Tatang RJ, Tsila HG, Wabo Poné J (2017). Medical important parasites carried by cockroaches in Melong subdivision.

[32] Isaac C, Orue PO, Iyamu MI, Ehiaghe JI, Isaac O (2014). Comparative analysis of pathogenic organisms in cockroaches from different community settings in Edo State, Nigeria. Korean J Parasitol.

[33] Martínez-Girón R, van Woerden HC (2014). Bronchopulmonary lophomoniasis: Emerging disease or unsubstantiated legend?. Parasit Vectors.

[34] Iboh CI, Ajang RO, Etta HE, Abraham JT (2015). Public health implications of cockroaches within households in Calabar municipality, Cross River state, Nigeria. Glob J Pub Health Epidemiol.

[35] Zakaria A, Al-Share B, Al Asad K (2016). Primary pulmonary amebiasis complicated with multicystic empyema. Case Rep Pulmonol.

[36] Liu YY, Ying Y, Chen C, Hu YK, Yang FF, Shao LY, Cheng XJ, Huang YX (2018). Primary pulmonary amebic abscess in a patient with pulmonary adenocarcinoma: a case report. Infect Dis Poverty.

[37] Wallace GD (1972). Experimental transmission of *Toxoplasma gondii* by cockroaches. J Infect Dis.

[38] Smith DD, Frenkel JK (1978). Cockroaches as vectors of *Sarcocystis muris* and of other coccidia in the laboratory. J Parasitol.

[39] González-García T, Muñoz-Guzmán MA, Sánchez-Arroyo H, Prado-Ochoa MG, Cuéllar-Ordaz JA, Alba-Hurtado F (2017). Experimental transmission of *Toxocara canis* from *Blattella germanica* and *Periplaneta americana* to a paratenic host. Vet Parasitol.

[40] Esposito WA, Chew GL, Correa JC, Chillurd SN, Miller RL, Kinney PL (2011). Quantitative measurement of airborne cockroach allergen in New York City apartments. Indoor Air.

[41] De Lucca SD, Taylor DJ, O’Meara TJ, Jones AS, Tovey ER (1999) Measurement and characterization of cockroach detected duringnormal domesticactivity. J Allergy Clin Immunol 104; 672-680 10.1016/s0091-6749(99)70341-6 10482845

[42] Cazorla-Perfetti D, Morales Moreno P, Navas Yamarte P (2015) Identification of Lophomonas blattarum (hypermastigia: cristomonadida, lophomonadidae), causal agent of bronchopulmonary lophomoniasis, in synanthropic cockroachs from the Coro university hospital, Falcon state, Venezuela. SABER. Revista Multidisciplinaria del Consejo de Investigación de la Universidad de Oriente 27: 511–514. (**Article in Spanish**)

[43] Rina S, Melva P, Geminis V (2015). Primer hallazgo de Lophomonas spp. (Metamonada, Lophomonadida) en la cucaracha doméstica (*Periplaneta americana* Linnaeus) en Panamá. Rev Arg Parasitol.

